# Fractal algorithms and RGB image processing in scribal and ink identification on an 1819 secret initiation manuscript to the “Philike Hetaereia”

**DOI:** 10.1038/s41598-023-28005-4

**Published:** 2023-01-31

**Authors:** Ion Andronache, Ioannis Liritzis, Herbert F. Jelinek

**Affiliations:** 1 Research Center for Integrated Analysis and Territorial Management – CAIMT, Faculty of Geography, University of Bucharest, 4-12 Regina Elisabeta Avenue, 3rd district, 030018 Bucharest, Romania; 2 Laboratory of Yellow River Cultural Heritage, Key Research Institute of Yellow River Civilization and Sustainable Development & Collaborative Innovation Center On Yellow River Civilization, Henan University, Minglun Road 85, Kaifeng, 475001 China; 3 European Academy of Sciences & Arts, St. Peter-Bezirk 10, 5020 Salzburg, Austria; 4 School of History, Classics and Archaeology, College of Arts, Humanities & Social Sciences, Dept of Archaeology, Edinburgh University, Edinburgh, EH8 9AG Scotland; 5 Department of Biomedical Engineering and Health Engineering Innovation Center, Khalifa University, Abu Dhabi, United Arab Emirates

**Keywords:** Chemistry, Mathematics and computing

## Abstract

Historical texts incorporate important characteristics that need to be assessed including genre, text structure and content. Often overlooked are characteristics of handwritten manuscripts commonly divided into legibility, readability and aesthetics. To determine the scientific feasibility of classification of handwritten texts an objective approach is developed to describe twenty handwritten pages of an 1819 Greek manuscript, that refers to the initiation to the Greek secret “friendly society” (Philike Hetaereia) organization, established as part of the Greek independence against the Ottoman Turks. It is investigated through a fractal and RGB image analysis approach. Fractal Minkowski Dimension was applied on the handwritten text and the RGB color analysis on the ink and paper and both were used as a non-invasive manner and revealed interesting results. The novel RGB image analysis and the fractal analysis of the manuscript identified respectively, five iron gall inks and four scribes from the ink content and handwritten styles, of the compact five lines text and whole text pages. The novel approach was verified with another old manuscript of known ink pigments, as well as with thirteen known handwritten texts of that period and four prints representing modern and similar period texts substantiating the findings of the novel methods.

## Introduction

In 1814 three Greeks met in Odessa and decided to set up a strictly conspiratorial organization – the “Philike Hetaireia” (friendly society)—which would prepare the uprising of all Greeks. According to earlier historians, these were Emmanuel Xanthos, Nikolaos Skoufas and Athanasios Tsakalov. All three had already shared revolutionary ideas and the Masonry fellowcraft related to Carbonarism (informal network of secret revolutionary societies active in Italy from about 1800 to 1831 most likely emerged as an offshoot of Freemasonry)^1^. Those who first conceived the idea of forming a secret group of Greeks to prepare an uprising for independence from the Ottoman rule in Greece were active in the then free World^2,3^. In the spring of 1821, the Society began the Greek War of Independence^4,5^.

*Philike Hetaereia* members had to be initiated by a procedure similar to freemasonry. The whole initiation included a written text with a confirmation letter (ephodiastikon) showing membership in the Friendly Society as well as the Catechism text. Such materials kept by its members are significant testimony to both the character of the organization and the qualities of its members. Upon entering the Friendly Society, the initiates offered a sum of money and a letter of dedication, where they noted their basic data and the coded initials with which they can be identified and correspond with each other^6^.

Writer identification still remains an active area in the field of handwritten document analysis and in color measurements of inks on a document coming from a different or the same source, with several attempts and results having been published in the past^7,8^. Certainly, fractal algorithms relate to the process of complexity and therefore can be applied to historical manuscripts such as written by *Philike Hetaereia* members^9^. Handwriting is complex regarding several aspects of style and size of letters, densely and sparsely writing and defines self-invariance^10^ (see below). Moreover, writing is often represented by (e.g.) splines and polynomials with Euclidean transformations^11–13^. The concept of fractal geometry was developed by Benoît B. Mandelbrot (1924–2010) based on previous pioneering studies by Jules Henri Poincaré (1854–1912), Georg Ferdinand Ludwig Philipp Cantor (1845–1918), Lewis Fry Richardson (1881–1953), Niels Fabian Helge von Koch (1870–1924), Waclaw Sierpinski (1882–1969), Karl Menger (1902–1985) and Karl Weierstrass (1815–1897). Fractal geometry was initially developed from the observation that shapes such as rivers, tree branching patterns, or internal organs such as the lung bronchi or blood vessels were not identified as Euclidean patterns^14^ as well as from the analysis of “monsters” of Euclidean geometry: mathematical or natural, phenomena not fully explainable or quantifiable through Euclidean geometry. Mandelbrot first used the term “fractal” in his book *Les Objects Fractals: Shapes, Hasard et Dimension*^15^ and later in The Fractal Geometry of Nature^16^ to designate continuous but indistinguishable objects or phenomena in the meaning of mathematical analysis. According to Mandelbrot, fractal objects are mainly characterized by four properties: irregularity of their shape, self-similarity of their structures, fractal dimension and scale invariance. In principle, a fractal is a pattern that repeats indefinitely, and every section of the fractal, no matter how zoomed in or out, seems to be identical to the entire picture.

The fractal character of an object indicates the existence of a morphological complexity. This complexity is expressed both by periodicity and by a similarity, a repetition of patterns on several scales: a structure in which each element is a similar structure made up of other similar structures, until a resolution limit. By dividing a fractal into two or more parts, fragments are obtained whose structure is by no means simpler than that of the whole and defines self-invariance^10^. Self-invariance can be self-similarity (when it is isotropic), or self-affinity (when it is anisotropic, depending on the spatial direction).

Ideal mathematical fractals retain their pattern and complexity no matter how much the dimensional scale of the pattern is reduced or increased at which they are analyzed. All real objects are, in fact, approximate fractals: they have “fractal properties” if they are analyzed on a scale other than a very simplistic one. Perfectly straight lines, perfectly flat, spherical or hyperbolic surfaces are practically absent in nature (especially in living organisms), and the “imperfect” aspect is the expression of the fractal character of the surrounding reality.

Unlike mathematical fractals, natural objects are characterized by irregularity and “rough” shapes that appear as very complex. There is a long list of natural objects that morphologically are obviously approximate fractals^17^: clouds, snowflakes, crystals, the outline of mountains, shores, aquifers, cauliflower and broccoli, vascular systems, aerial and underground parts of trees, the organization of fungi, algae, lichens, corals and more. Many natural or technical processes have a fractal dynamic, a character more accentuated as the nonlinearity of the equations that can shape them become more pronounced. The best-known example are meteorological phenomena, but most physiological processes, and in general biological ones, recorded accurately and without filtration that retain only strictly periodic components, show fractal characteristics^18^. Already established applications of fractal analysis include population dynamics, epidemiology^19^, OCR for Arabic scripts^20^, architecture^21^, analysis of the tendency to concentrate economic activities in the main core of the national polycentric network^22^, pattern analysis in the creative economies in Romania^23^. Further application of the box-counting fractal analysis method for determining the compaction pattern of forest areas^24^ and analysis of the fragmentation tendency of forest areas in of Romania^25–29^ have been published. Kinetics and dynamics of animal behavior^30^, genome organization^31^, neuroscience^32^), works of art^33^ and many other examples highlight the presence of Euclidean forms and dynamics.

Fractal analysis is the set of methods of organizing, processing, and interpreting information about the complexity of objects. The fractal dimension is a ratio that provides a statistical index of complexity, comparing how the details in a fractal model or objects change with the scale at which they are measured. It is thus a measure of the capability of the objects to fill the space. Therefore, the fractal size does not have to be an integer number^34–36^.

### Why fractal Minkowski?

To analyze the shape of some object’s different fractal analysis methods can be used. The most common methods are: Ruler Dimension^37,38^, Perimeter-Area Dimension^39,40^, Gaussian Convolution^41^ or Minkowski Dimension^42^. Ruler Dimension and Gaussian Convolution are not suitable for the current analysis because they can only analyze open curves and handwriting mostly involves both open curves and closed curves. Moreover, both Ruler Dimension and Gaussian Convolution analyze only a single curve and a word, or a group of words do not fall into this limitation. Perimeter-Area Dimension allows the analysis of complex curves but can only analyze a figure, in our case a word or part of a word is not identified as a figure and hence these analysis methods are not suitable. The Minkowski Dimension does not have these shortcomings and hence was applied in the current study.

### Current methods for ink and scribe identification

Various archaeometry methods, invasive or non-invasive, for pigment identification in art and archaeology are known^43–45^. Inks of old manuscripts have been analyzed and characterizations of the different inks, are made by means of fingerprints. Inks may be black (iron gall (Iron-gall ink was produced by combining iron sulphate (FeSO_4_) with tannic acid (C_6_H_2_(OH)_3_COOH), which is typically derived from oak galls, which are tumor-like structures on oak trees.) or carbon), red (vermillion, Pb-based), blue (indigo) or another color derived from inorganic or organic based compounds. The pigment analysis is usually by various spectroscopic techniques. For example, Raman spectrum which indicates peak frequency characteristics of the respective compound^46–49^. X-Ray fluorescence (XRF) where the major chemical element indicates the respective compound (e.g. iron for iron gall; Bromide for indigoids group)^50^.Chromatography for royal purple from shellfish purple, also known as Tyrian purple in *Murex trunculus*^51–53^, Fourier transform infrared (FT-IR) spectroscopy (attenuated total reflectance (ATR) and transmission) spectra for organic binders and some inorganic elements either based on the stretching vibration peak of triple carbon (C≡C)bonds in carbon black and the characteristic peak of C–H or the Iron (II) sulfate bonds and other ingredients with different chemical bonds, compared with other techniques, such as scanning microscopy and gas chromatography combined with mass spectrometry, gave good results and corroborated infrared spectra findings^49,53^. Moreover, discriminating iron gall inks has been addressed by correlating their infrared (IR) spectra in liquid and dried states with the materials used in their formulations and considering their possible interactions^54^. Hyper spectral image (HSI) of handwritten notes can discriminate between inks that are visually similar in appearance^55^), though this method fails to segment inks correctly when applied to RGB scans of documents, since the inks are very hard to distinguish in the visible spectral range. In fact, Khan et al.^55^ collected RGB scanned images at resolutions 150 and 300 dpi made using a flatbed scanner and the local threshold binarization method of Sauvola and Pietikainen^56^. Their hyperspectral imaging by feature selection (HSI-FS) for a 400–720 nm range on three segmented ranges provided complementary information to distinguish inks and was more effective than RGB. However, even with applying feature selection results are inconsistent, most likely due to the implementation of the algorithm which is discontinued at a local maximum and therefore does not further explore additional bands for improved accuracy.

This short-coming of ink identification was overcome by applying in our study a new image pre-processing of images analysis and transformation to a binary image following six steps in order to improve the methodology of extraction and identify a specific color from the RGB image (see “Pre-processing forink color”). In particular, the binarization stage in our new method is an intermediate step not the final one, as in Khan et al.^55^.

For first time it is applied with a satisfactory outcome in distinguishing inks of the Greek manuscript but also for other cases. The clustering based on RGB images despite the claim of Khan et al.^55^, that it is unable to group similar ink pixels into same clusters, at least for RGB of colored images from archaeological ceramics has been shown to have a sound result^57^.

Other spectroscopic and handwritten identification studies have shown either the advantage of hyperspectral imaging compared to traditional color imaging, or for RGB images the images captured from non-visible bands. For the task of ink separation using the distance-based classification method for manuscripts in the National Library of Oslo^58^, and in Old Greek historical manuscript collections which are written in lowercase letters and originate from St. Catherine’s Mount Sinai Monastery, where a segmentation-free, fast and efficient technique for the detection and recognition of characters and character ligatures has been proposed^59^.The crucial element in the segmentation approach is to separate individual characters from a scanned bitmap representation of a page. Many strategies for segmenting handwritten text and numbers have been presented by Lu and Shridhar^60^.Lu and Shridhar discussed the various ways for segmenting handwritten characters, and Xiao and Leedman^61^ proposed a segmentation method based on handwriting knowledge, whereas Plamondon and Privitera^62^ proposed a segmentation method that partially simulates the cognitive-behavioral process used by humans to recover the temporal sequence of the strokes that comprised the original pen movement. Alternatively, more global approaches avoid character segmentation, looking at words as entities and using statistical methods to classify word samples^63^.

In general, several efforts for ink differentiation and classification in old manuscripts applying spectroscopic and algorithmic approaches have been reported and this is an ongoing field of research^64–66^. Similarly, some spectroscopic, chemometrics and algorithmic methods have been developed to spectrally characterize paper pulp (handmade or industrial)^67–71^.

On the other hand, the study of handwritten contents, whether they be textual or pictorial, is the subject of the subfield of pattern recognition and document image analysis and recognition known as handwriting recognition. Handwriting recognition has been reported in the past for various languages^72–78^.

The present work presents for first time the fractal investigation of a handwritten document which is the property of the Grand Lodge of Greece in Athens which consists of a complete text of an initiation to the Secret Society (*Philike Hetaereia*)^79^. To pave the way for developing safe, timely and an inexpensive methodology, tailored as much as possible to the manuscript of interest, this paper introduces novel approaches and advances that concern our understanding of the characterization techniques of iron-gall inks, and scribes’ style of manuscripts of the historical period. The aims of the research were met through a novel investigation combining analytical effort of non-invasive techniques, by using fractal Minkowski Dimension for text images and text lines and RGB color analysis for ink and paper, both of which function at the end work in a complementary manner to achieve the objectives. The aim was to examine (a) the number of scribers from the handwritten style of the concerned folio, (b) the identification of inks used, (c) the similarity of paper pages used to write the manuscript and (d) verify the novel methodology on well-known handwritten and print texts and inks of the past 400 years data.

## Results and discussion

### The five lines and full pages fractal analysis

The process of D_M_ estimation in these two cases has been thoroughly examined. The criteria used for decisions made of same/different writers are based entirely on the differences of D_M_ estimations because Minkowski algorithm are sensitive in all following five combined parameters and conditions (hence the complexity nature of the issue) in the writing style of scribes; Parameters: (a) the blank space between lines is a writing style of the scribe, and for larger distance the lower the D_M_ is and inversely (e.g. for minimal distance or touching the lines the D_M_ tends to 2), (b) the space between words. In fact, for (a) and (b) in binary form, the space of letters size occupied in the blank space, in our case of Fig. 12, ranged between 73–93%); Conditions: (c) different letter shapes are not mathematical fractals, (d) analyzed text (a number of lines) has same length per page text, or else any comparison with full length page may misleadingly assign a different scribe. In such semi full written pages, we isolate similar length and distance of lines for comparison, and for the validation of the result, (e) the standard deviation (SD) of the estimated Minkowski dimension should be kept low; in the Minkowski analysis of our images, SD is between 0.01 to 0.02 and Rsq = 0.990–0.999, and such SDs make D_M_ estimated values comparable (for generated fractals the SD is 0.001 and Rsq = 0.999). It is important that SD is very small and Rsq close to 1 (more than 0.99) for the fractal size estimation to be validated. Higher values of SD or Rsq below 0.99 indicate that the D_M_ estimates at different scales (dilating by 1 pixel, then by 2 up to 10 pixels) give different results and do not fit into the philosophy of part of image equals the whole image, which characterizes objects with fractal properties.

**Figure 1:**
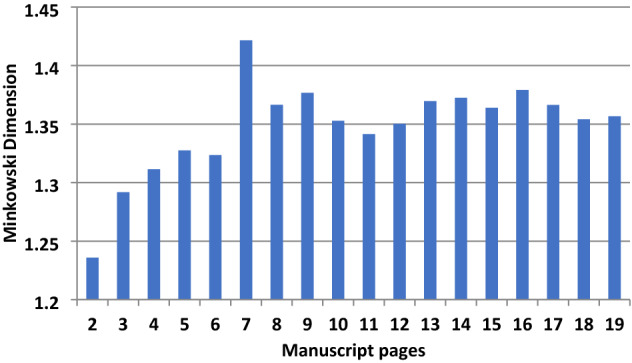
The 5-lines analysis per page from page 2 (confirmation letter) to page 19 of the manuscript.

Following the 5-lines analysis (Fig. 1), in the writing style on page 2 of the manuscript, the Minkowski dimension (D_M_ = 1.23 ± 0.011) was found to be more that 1SD different from pages 4–6 (D_M_ = 1.31–1.32), and from page 3 which has D_M_ = 1.29 ± 0.017 and from pages 7–19, which have the highest fractal dimension (D_M_ > 1.34). In fact, within 1SD the average D_M_ for 4–6 pages is 1.32 ± 0.008 and for 8–19 is 1.36 ± 0.01. This implies **four scribes** contributed to this manuscript.

**Figure 2:**
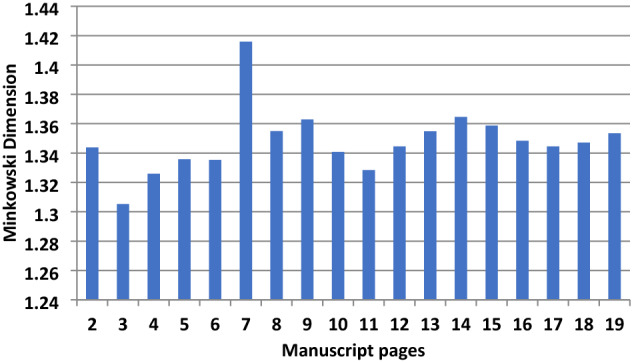
All lines in one page fractal analysis.

Figure 2 shows results of the full pages from page 2 to page 19. The trend is comparable to the 5-lines per page analysis (Fig. 1) but with some particular attention to what concerns the satisfaction of the conditions we mentioned above. Pages 2 (half written and record the recommendation letter) and 7 (cryptographic alphabet sparsely written page) justify a high and out of comparison scale. Pages 4, 5, 6 have an average D_M_ = 1.32 ± 0.005 and indicate **one scribe**. Whilst page 7 has large unwritten spaces. Moreover, the pages 8 and later have D_M_ = 1.35 ± 0.01 indicating **another scribe**, the apparent fast style in the writing did result in the binary mode representation of letters for similar words to have a slight variation impact on the fractal analysis outcomes which nevertheless does not preclude true differences. Hence, the writing is attributed to the same scribe. Hence, due to the incomplete handwritten pages, at least **two scribe**s are proposed for all written pages which surely is not representative for the reasons explained.

Therefore, a change in script D_M_ metrics theoretically does not necessarily mean a change in hand but that all criteria are not met. In *Philike Hetaereia* the parameters (a) and (b) and conditions (c–e) are met with more determinable being the (a) and (b), and the differentiation between scribes is thus accepted. Indeed, the proportion between black pixels and all pixels (white and black) for the 5 lines analysis ranges between 90.7 to 94.6% (part of cleaned extracts in binary form is shown in Fig. 17b). For example, pages 2 and 7 though have ~ 94% black pixels but the D_M_ is very different being 1.23 and 1.42 respectively. The latter is another reinforcement for these pages attributed to two scribes.

The present method and applicability for identifying scribal has been verified on printed text of English, Romanian and Russian literature and shown in the next Sect.  “Confirmatory data analysis”.

### RGB values of inks and paper

**Figure 3:**
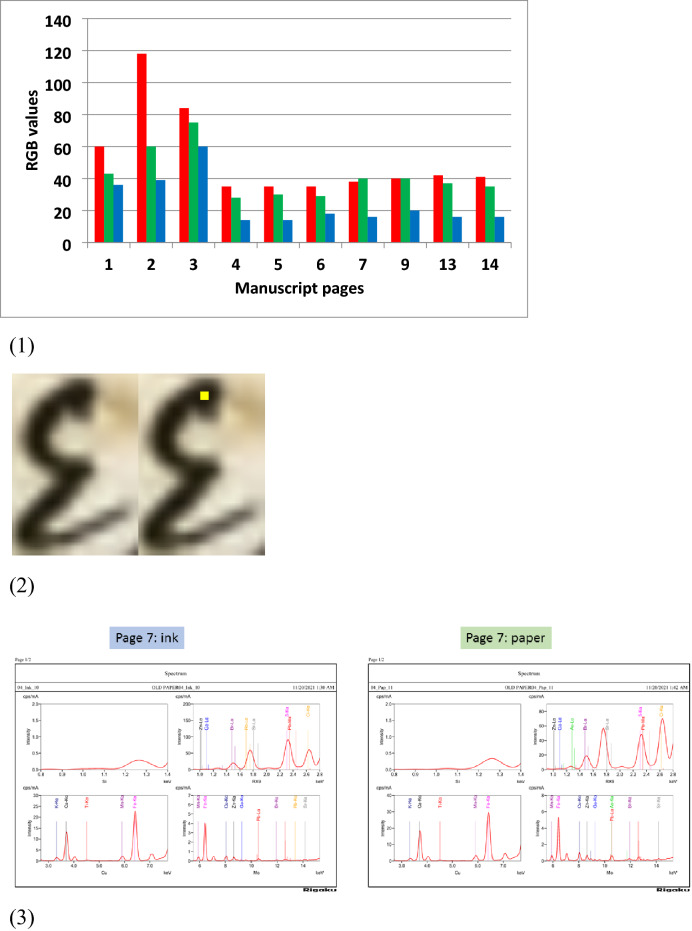
(**1**) RGB histogram of ink from the 10 pages of the manuscript. (**2**) measured pixels in the yellow region of interest (ROI) on a letter is given for letter (epsilon) ε; (**3**) XRF spectra of ink and paper of page 7. Note X-rays peaks with relative intensity. Pages are mentioned according to the numbering of the pages in the real manuscript. Si, RX9, Cu and Mo indicate the different type of secondary targets used by the NEG CG apparatus breakthrough concept, in order to realize a polarized optical system which reduces considerably continuous X-rays, thus enabling higher sensitivity and precision than ordinary ED spectrometers.

Ink color was examined from 10 selected successive pages (1 to 14). The analysis indicated that the ink color of the first page (front cover) is different from the remaining pages. Although the fading of the first and other pages is a likely occurrence for ageing documents, the observed difference is supported also by different paper material of first page (similar to the back cover) compared to the following ones. Moreover, the handwritten manuscript was exposed to similar environmental conditions and oxidation from the inks, hence all pages are expected to reflect a representative and comparable status of their RGB values. In fact, this is supported by the visually similar ageing due to degradation, while some severe and accidental moisture attack are apparent and avoided. The cover and back pages also seem a bit thicker. The threshold of attributing level of similarity is based on the averages of each colour channels and their respective summation within 1SD. Inks from pages 1, 2 and 3 differ from each other and from the other pages analyzed. Inks from pages 4, 5 and 6 are similar (averages R = 35 ± 0.01, G = 29 ± 1, B = 15 ± 2.3 and sum 79) and form another ink and pages 7 to 14 (averages R = 40 ± 1.7, G = 38 ± 2.4, B = 17 ± 2, and sum 95) another one, having close RGB values within their individual colour channels and their RGB summation. To determine the color attributes of the writing from an RGB image of inks, three separate images corresponding to red, green and blue values were extracted. Each of these images has integer values from 0 to 255, where 0 corresponds to the darkest saturated value and 255 corresponds to the highest saturated value of each of the R, G, B values. Thus, according to this RGB analysis **five (5) different inks** may have been used in these pages of the manuscript (Fig. 3) (the five different black inks does not imply scribes). An example of measured pixels in a ROI is given for letter (epsilon) ε (Fig. 3). The inks in the studied manuscript are iron gall^80^. In fact, selected spots on representative pages have been analyzed by means of XRF/EDS using a NEX CG, Rigaku system, with an X-ray tube with Pd anode with a tube power of 50 W (50 kV to 2 mA). It is equipped with 5 secondary targets and a Silicon Drift Detector (SDD). Standardless analysis was performed using RPF-SQX (Rigaku Profile Fitting – Spectra Quant X) software with a fundamental parameters method for accurate quantification combined with full profile fitting method^80^. Iron content in pages varied between 5–32% (see a representative spectrum in Fig. 3). Variations in iron content reflects the ink recipe and the starting raw materials, i.e. indicates the iron gall ink type but also the ageing and batch (lot) that the portion of the inkpot used by a scriber derives from. In addition, iron gall inks before 19th c., have much less carefully formulating recipes^81,82^.

**Figure 4:**
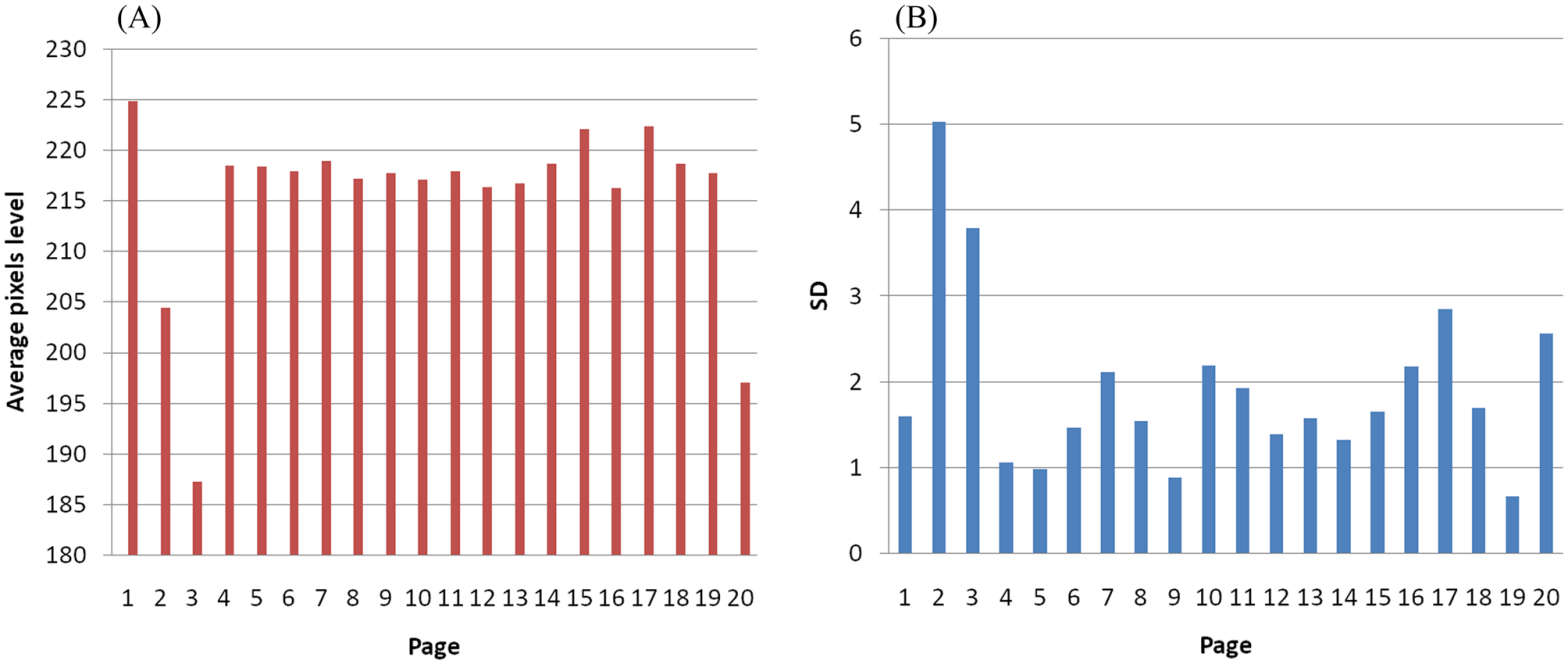
(**A**) Histogram of RGB for paper from pages 1 to 20. A 40x40 pixels areas per page have produced the result that indicates obvious differences in certain pages beyond the standard deviation. (**B**) standard deviation per page.

Within these paper pages the dominant part of each blank page without writing or visual degradation was also selected and an RGB histogram was determined. From the histogram the pixel in the dominant class was selected as a percentage (mean of histogram). Figure 4 provides the R, G, B results for the blank parts of the pages analyzed when taking two readings per page at the top (a) and bottom (b).

A verification test on ink identification with the present approach is shown in the Sect. “Confirmatory data analysis”, for other manuscripts that are several hundred years older and analyzed by Raman spectroscopy.

A similar explanation to the above for inks applies to the paper analysis. It is the enhanced pre-processing that resulted in a more representative ground truth RGB image analysis of paper characteristics. It is expected that any differences in color between the pages in color of the paper may reflect alterations due to environmental factors which the paper was exposed to over time such as exposure to humidity, light sources, and oxidation from the inks. Yet all 20 examined pages are subjected to the same factors (similarly, the 16th and 17th c. ones dealt with in Sect. “Confirmatory data analysis”). All pages are subjected to same environmental conditions as mentioned above. Moreover, from each page ROI of 40x40 pixels was chosen visually as white as possible and the consistency of the results suggest the paper material was from the different production technique and ingredients used at least for pages 1, 2, 3, 4-19, 20 beyond standard deviation. This conclusion was reached as the color of the pixels within a page varied in a similar pattern as between the different pages and in comparison, to confirmatory data analysis, for other manuscripts that are several hundred years older (see Sect. “Confirmatory data analysis”). These papers of the older manuscripts are of different origin and technique/material and as expected they differed compared to our Fig. 4. According to the watermarks, the first handwriting associated with the scribes of the manuscript was written on different paper than the second and third handwriting. Each of the examined manuscript and those in the confirmatory section have been subjected to different preservation conditions. But the analysis of any one manuscript includes the same impact to all elements for every factor when compared for each single case.

## Confirmatory data analysis

The study analysis of 16th and 17th century manuscripts which bear inks of various types identified as ground truth by spectroscopy was a good example to test our new RGB method for ink identification. These manuscripts were chosen only for their inks not for their handwritten analysis.

The 13 manuscripts chosen date around the examined manuscript age and a later one in order to show the differences in the D_M_ amongst different known scribes and also for testing the D_M_ of same authors (Koraes and Ypsilantis), but test the different paper material production.

### Identification of inks

**Figure 5:**
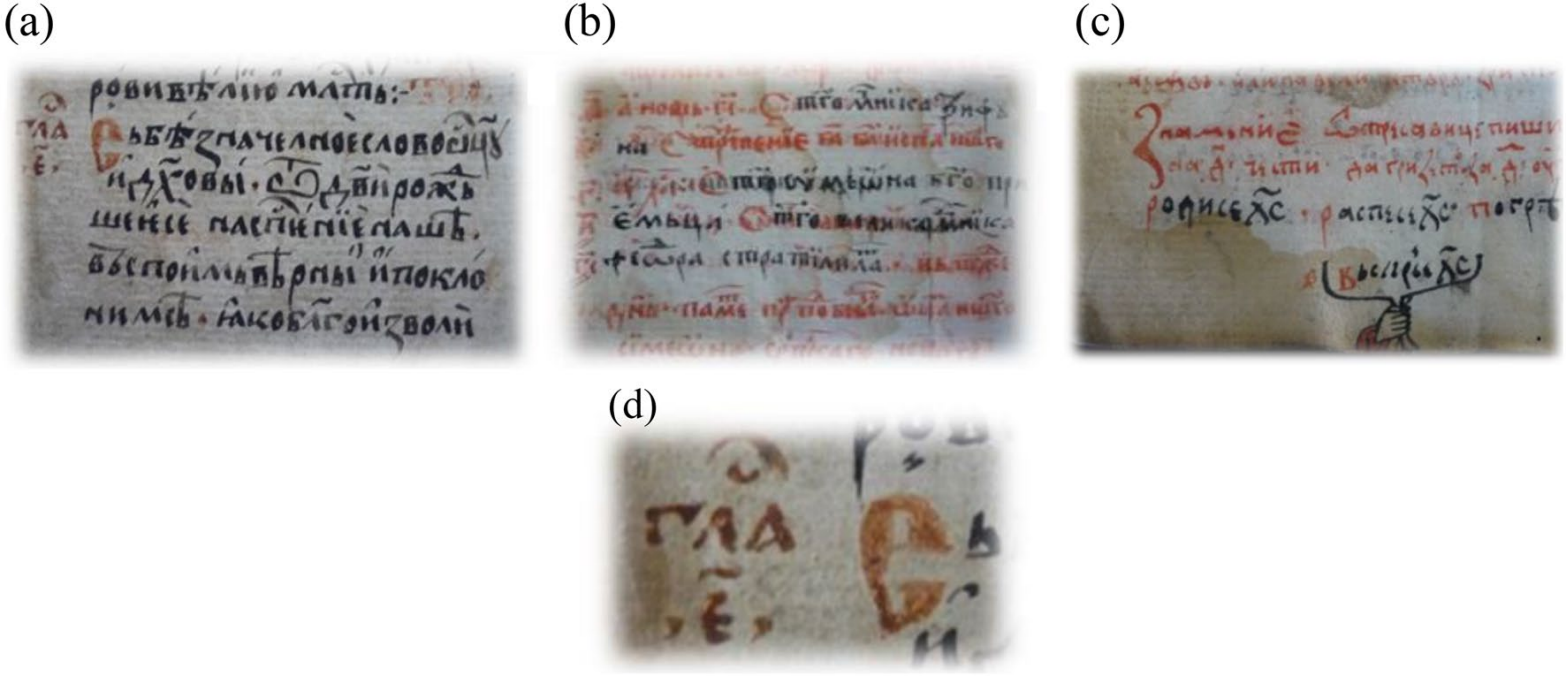
Collection of scriptures of, (**a**) first handwriting with black and red ink, sixteenth century, (**b**) second handwriting with black and red inks, seventeenth century, (**c**) black and red inks of third handwriting of seventeenth century, and (**d**) the upper left detail of lateral guides and initial letters of dark and pale red of (**a**)^46^. (photo permit by principal author of article 46, Dr Kostadinovska, M).

A verification test of the satisfactory separation of scribal with RGB images of letters of different ink color shown with the studied manuscript with another earlier manuscript (sixteenth-seventeenth centuries) from the Slavic literature is presented. Kostadinovska et al.^46^ analyzed pigments and inks in situ using micro-Raman spectrometer, of a manuscript dated back to 16th century (1575–1600) currently stored at the Historical Museum of Kruševo, Northern Macedonia, Skopje. It is recorded by the National and University Library “St. Clement of Ohrid” in Skopje under the reference number (signature) IMC Ms 2. The manuscript is a Liturgical Collection of chronicles, scriptures, and other material that contains 98 folios (14 × 9 cm) bound in a leather cover and written in Old Slavic language with three handwritings (Fig. 5). The first handwriting dates from the fourth quarter of sixteenth century, while the second handwriting and the third handwriting are from the late seventeenth century (1680–1700). According to the watermarks, reported by the original authors Kostadinovska et al. (2013)^46^, the first handwriting was written on different paper than the second and third handwriting.

The three texts of sixteenth and seventeenth centuries with black and red inks are shown in Fig. 5named as 1A (of 16th c.), 1B (of 17th c.), 1C (of 17th c.) (maintaining same numbering of original^46^ publication that is Nos 1, 2, 6, 9).

#### Sixteenth century 1st handwriting example

The analysis of 1A-B (16th c.) refers to the black ink in the first handwriting and shows the presence of iron gall ink; the 1A-R refers to the red ochre and red lead, with the addition of a third pigment vermilion (HgS); the 1A-DR refers to the dark red as red ochre or hematite (Fe_2_O_3_) and red lead or minimum (Pb_3_O_4_).

**Figure 6:**
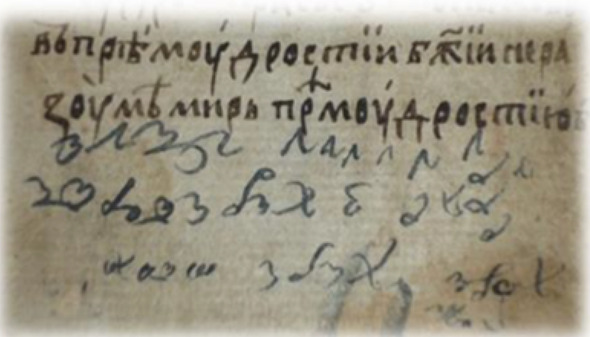
Blue ink of indigo and black ink of the first handwriting scribe (photo permit by principal author of article 46, Dr Kostadinovska, M).

The No 2 (Fig. 6) has two writing examples, a black and a blue indigo of the1st handwriting (16th c.) with the 2B of iron gall ink and the 2BL for blue indigo.

**Figure 7:**
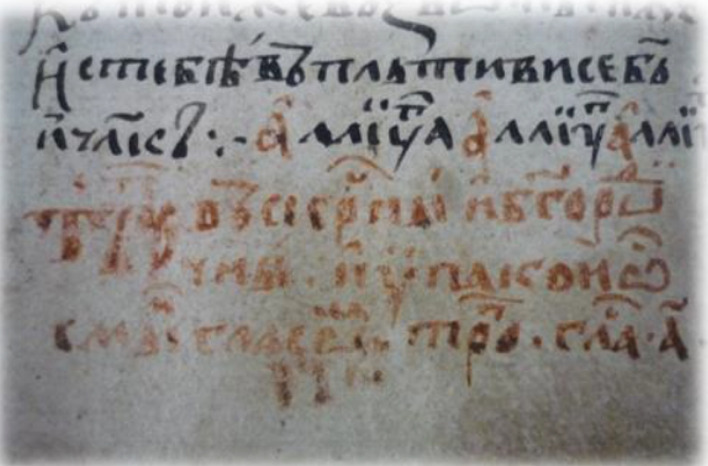
Degradation of red ink in the first handwriting (photo permit by principal author of article 46, Dr Kostadinovska, M).

Another handwritten text (No 6 in Fig. 7) by the same authors^46^ with red and black of the sixteenth century was analyzed with 6B for the iron gall ink and 6R for the lead-containing pigments (such as lead white and red lead).

**Figure 8:**
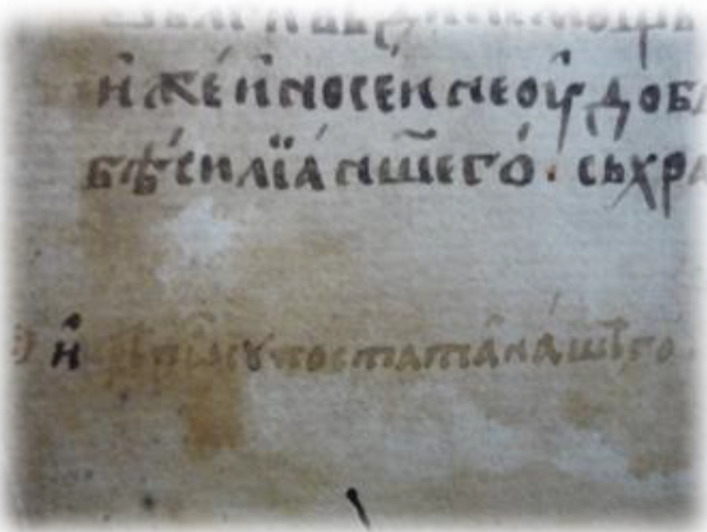
Parts of the folio of the first handwriting in which fading of black iron gall ink is present(photo permit by principal author of article 46, Dr Kostadinovska, M).

Another text is the No 9 (Fig. 8) with black iron gall (9B) and fading black (9FB) ink of the first handwriting in the16th c. Visual inspection of the first handwriting reveals a fading of the iron gall ink), although there was no visible evidence for its corrosion.

#### Seventeenth century 2nd and 3^RD^ handwriting

The No 1B (Fig. 5) is with red (1B-R) and black carbon (1B-B). The 1B-B black carbon is a soot-based pigment from mineral or plant not animal -bone/ivory origin^46^. The red 1B-R is Vermilion (HgS) which was the only red pigment identified in the text and ornamentation of the second and third handwriting (Fig. 5b,c). Its natural mineral, cinnabar, was widely used by ancient scribes because of its availability and simplicity for its preparation. Structural and spectroscopic techniques cannot distinguish between cinnabar and vermilion since they are chemically identical. The next inks of the seventeenth century text are the third handwritten example using black carbon 1C-B and red vermilion 1C-R (Fig. 5c).

### The RGB image processing results

**Figure 9:**
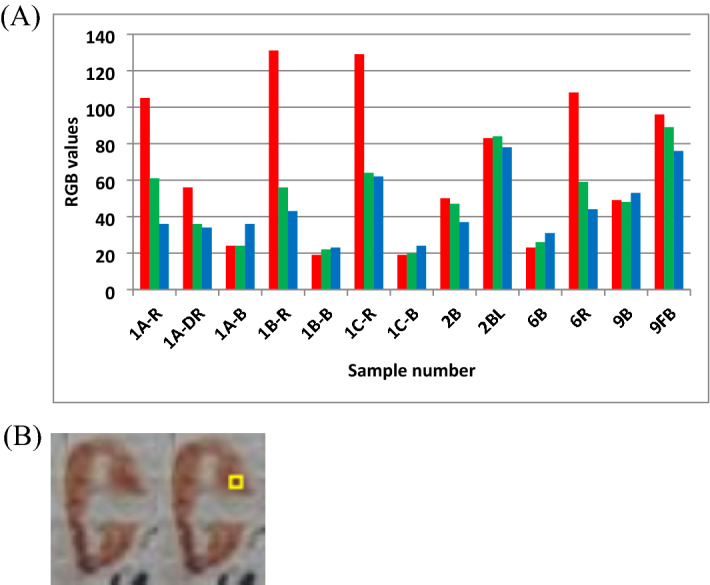
(**A**) RGB per ink in respective folios [Black inks: 1A-B (16th c., iron gall ink), 1B-B (17th c., black carbon), 1C-B (17th c., black carbon), 2B (16th c., iron gall ink), 6B (16th c., iron gall ink), 9B (16th c., black iron gall), 9FB (fading black ink); Red inks: 1A-R (16th c., red), 1A-DR (16th c., dark red); 1B-R (17th c., red); 1C-R (17th c., red vermilion), 6R (16th c., lead-containing red), and, 2BL (blue indigo)], (**B**) Letter C measured four pixels in the yellow ROI.

The results of the RGB methodology applied in the image processing produced very encouraging information regarding identifying scribal in a handwritten manuscript (Fig. 9a). In any case the decision made regarding similarity of inks is based upon R, G, B investigatory values using IQM on segmented regions of interest (ROI) on the letter. The ROIs per letter is chosen the parts that ink is well preserved. The final obtained R, G, B values in this way varied less than 1SD (see above “RGB values of inks and paper”). Within such a variability the similarity/difference comparison between inks is asserted (see Fig. 9b).

The *black carbon* of the 17th c. writing ink named 1B-B and 1C-B are similar. Both are lower in luminosity intensity from 1A-B and 6B of 16th c. handwriting using *iron gall black ink*. But the iron gall black ink of 9B is higher in luminosity intensity almost doubled from iron gall 6B. Thus, the four black inks from carbon and iron gall as a first approximation are distinguishable. Yet the intensities between iron gall inks may be related to the excessive ink spill observed at the analysis point. In this case the difference in ink quantity on the paper needs to be representative (which can be important depending on the moment when the quill pen (It is the hollow shaft of a feather.) was filled in the inkpot). The colour of the ink changes depending on when the scribe last dipped his pen and so the difference in ink quantity on the paper can be compensated by selecting some ROI using crop function in the ink region avoiding the faint or non-uniform colour.

The red ink 1A-R of 16th c. is of the same order with the dark red 1A-DR (16th c.) and the slight difference due to fading or less ink quantity used; the latter being most probable when observing the image. These red inks are similar to both 1B-R and 1C-R of the 17th c with slight differences in intensity rather due to fading. Both the red vermilions 1B-R and 1C-R of 17th c. are very distinct and like 6R lead-containing pigment of 16th c. but 6R has a lower red component (108 versus 130) with respect to vermilion inks, offering a possibility to separate the two different red pigments. Note that this lead-based red pigment was in contact with air for some time and the presence of bacteria and fungi is that the original color pigments we reconverted into lead sulfide (PbS)^47,48^. This could explain the darkening of the red ink observed in the first handwriting. More work on vermilion and led-based red pigments is therefore needed. The blue indigo 2BL is quite different in RGB from all other color inks, as well as the faded black 9FB.

The same ink and/or any damage of the paper surface as well as fading of the original ink is explained in Sect.  “Identification of inks”. The excess of iron ions stimulated the processes which favored the breakdown of cellulose, leading to the destruction of the paper surface as well as fading of the original ink^83,84^.

No fractal analysis is used for RGB of ink and paper. The confirmatory data are evident in Fig. 9 and explained in the following text for any similarity or slight difference which is also visually recognized.

### Scribal identification

#### Four modern and one nineteenth century printed texts

**Figure 10:**
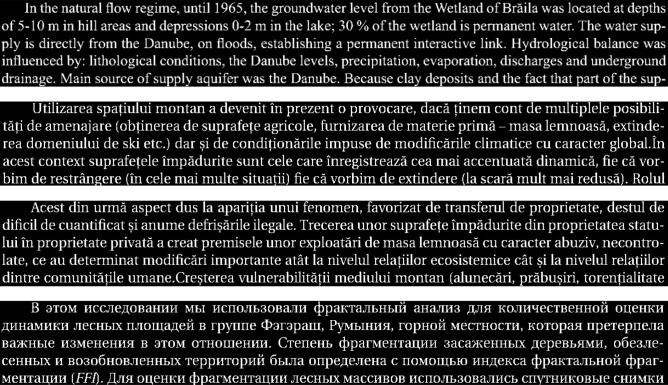
The five lines’ texts in English, two Romanian and one Russian.

**Figure 11:**
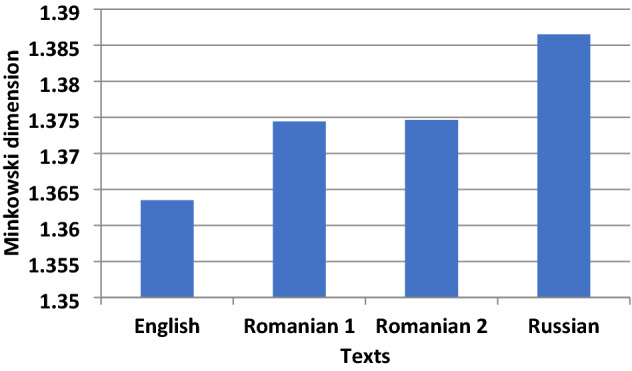
Minkowski fractal dimension of the four texts of Fig. 10.

To prove the validity of our methodology including the abovementioned (Sect.  “The five lines and full pages fractal analysis”) parameters and conditions in identification of different letter types observed in handwritten texts by different scribes of same alphabet we tested the methodology on four images written in three languages. Four texts of five rows – one in English, two Romanian, one Russian were analyzed (Fig. 10). All the modern four extracts are of similar fond type (Times Roman) and size and lead to interesting results. Figure 11 is the plot of the respective Minkowski dimensions. The writing examples of Romanian and English are close within 0.01 (D_M_ = 1.37 and 1.36 respectively) but quite different from the Russian (1.39). The similar D_M_ is because the Romanian and English alphabets are of similar shape Latin letters, and a slight difference between them is due to the diacritics in Romanian. On the other hand, the Russian alphabet is not Latin but Cyrillic with diacritics and moreover, Russian has 20 different latter shapes from English and Romanian. This confirms our expectation of Romanian close to English but not to Russian. Certainly, all four of them are different from the 1801 printed extract (12-NOM) with D_M_ = 1.30 (see Fig. 13 below).

### Thirteen handwritten texts of the beginning of 19th and 20th c

**Figure 12:**
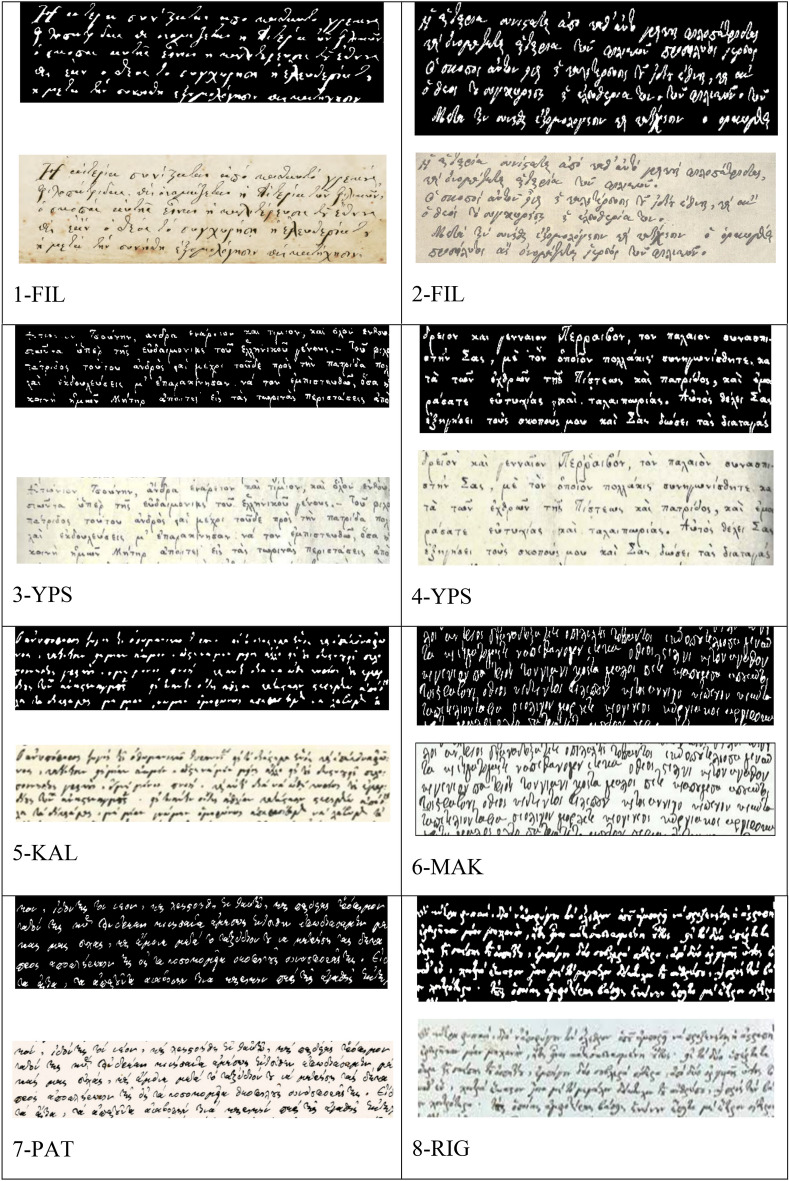

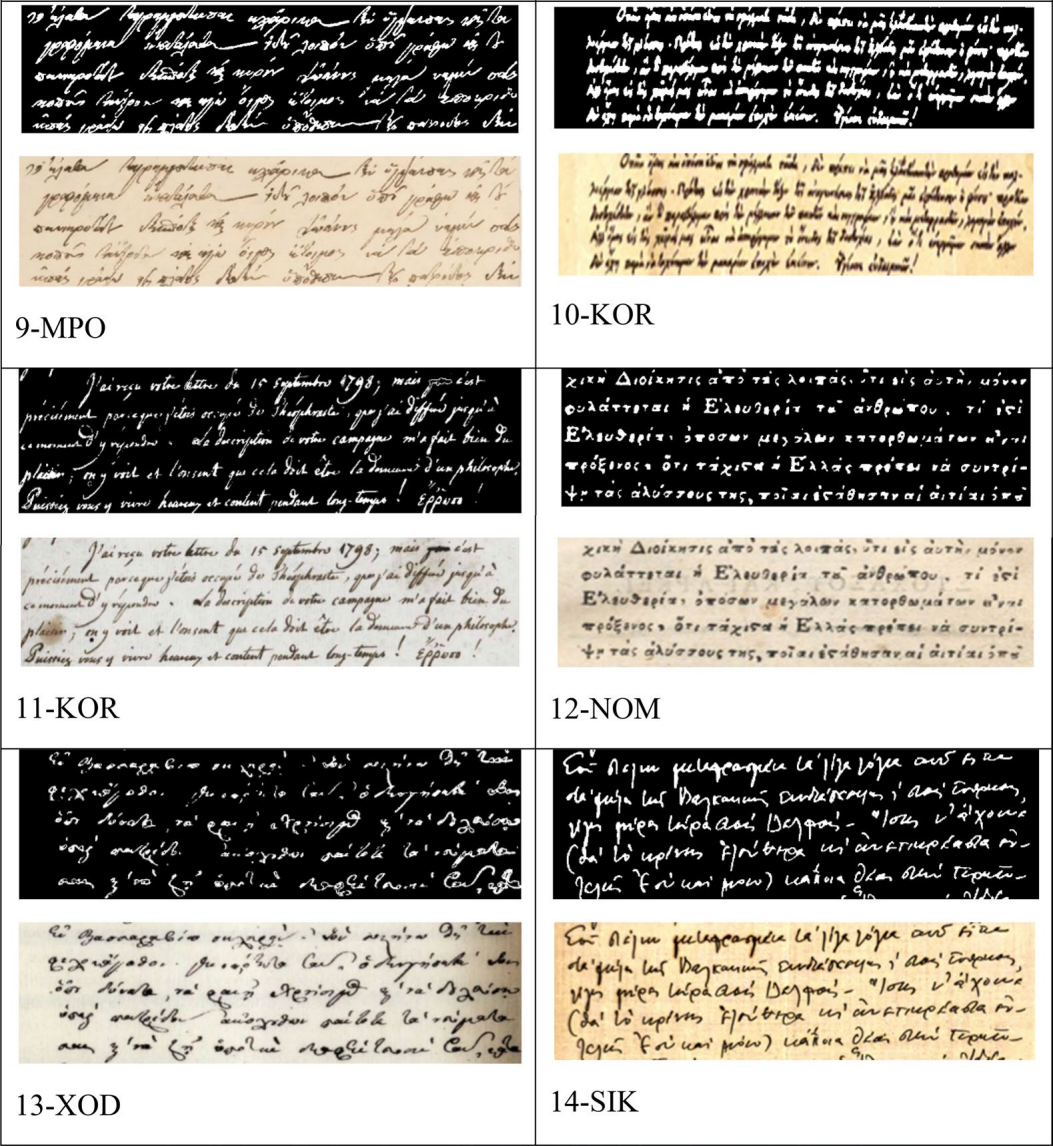
The fourteen RGB and binary images of the test handwritten extracts from letters including a printed version of 1801 (12-NOM). The sources of images are described in Table 1.

**Figure 13:**
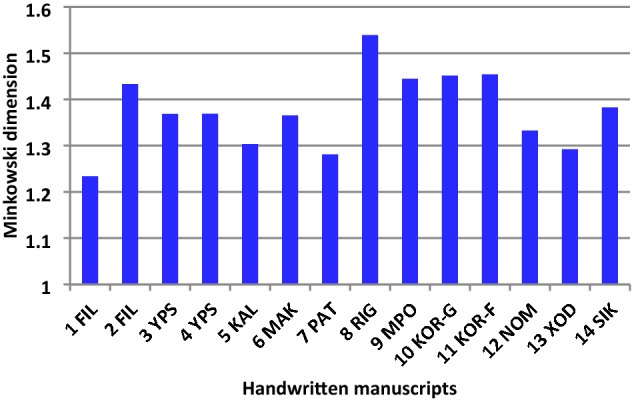
Minkowski fractal dimension of known authors handwritten manuscripts.

Thirteen selected handwritten texts with five lines originated around the same period of the studied manuscript; in fact, 12 are from the beginning of nineteenth century, and one from 1930; the latter as an extreme example of a manuscript stemming from hundred years later than 1821 (Table 1, Fig. 12). In all cases there isa clear distinct difference between location but not for the same authors (10-KOR and11-KOR for Koraes example, D_M_ = 1.45 and 3-YPS and 4-YPS for Ypsilantis, DM = 1.37). We note the two extracts of *Philike Hetaereia* manuscripts, the present and another one, with the same content on the aim of the society and wording, have different fractal dimensions (1-FIL and 2-FIL) as expected. Of interest is the unique style of the Rigas with the highest fractal dimension (D_M_ = 1.54) (Fig. 13).

**Table 1:** The fourteen handwritten manuscripts from ca.1790–1930 (all in Greek and one in French).

Code name	Source
1-FIL	FIL-OURS-AIM (1819) From Pythagoras (2016) Journal, extract regarding the aim of the initiatory process
2-FIL	FILIKI -AIM- OTHER, ca.1820, https://clioturbata.com/%CE%B1%CF%80%CF%8C%CF%88%CE%B5%CE%B9%CF%82/chatzopoulos-filiki-eteria/(anotherextract regarding the aim of the initiatory process)
3-YPS	3-Ypsilantis, «HierosLochos» 1820, http://www.askiweb.eu/images/2_aiones_se_21_ekpompes/61821Small.pdf; Alexandros Hypsilantis, 1792–1828,
	Greek patriot and revolutionary leader.Descended from a royal Phanariot family that ruled Wallachia during the Ottoman yoke. He served as a senior officer in the Imperial Russian Army in the Napoleonic wars, was prince of the Danubian Principalities and was one of the leading members of the Society of Friends (PhilikiHetaereia),
	He formed an army of 500 young volunteers that comprised the “HierosLochos” (Sacred Band). His written speech to the Greekrevolutionary
4-YPS	4-Ypsilantis, Ismail (near Braila, Romania) 1820,opening trumpet by Hyspilantis to the chiefs of the Greek armies, in Ismail Romania,for the revolution; http://www.askiweb.eu/images/2_aiones_se_21_ekpompes/61821Small.pdf
5-KAL	Kalamata 23 March 1821,handwritten text, the letter of the chiefs of the Messenian senate in Kalamata to the European common opinion that the Greeks rose up for their freedom; https://www.arcadiaportal.gr/timeline/apeleytherosi-tis-kalamatas-dimioyrgeitai-i-messiniaki-geroysia
6-MAK	Μakrygiannis, 1843, handwritten extract from the Memoirs of Makrygiannis, whichis perhaps the only extensive sample of a fighter's text from 1821 that hascome down to us without passing through the filter of some literate mediator;https://www.pancreta.gr/book.php?p=20270
7-PAT	Patriarch Gregorios E’ to Ioannis Varvakis, 1821, handwritten thanks letter for a considerable amount of money donated by Varvakis for "the establishment of a common shrine box in favor of the poor. https://www.elculture.gr/blog/article/sto-fos-agnosta-ntokoumenta-gia-tin-elliniki-epanastasi-kai-ti-filiki-etaireia-mesa-apo-ta-archeia-tou-mouseiou-faltaits-sti-skyro/
8-RIG	Rigas, 1790, handwrittenRigas ‘work «Physicsanthology», 1790 Vienna (re-edited by Scientific Society of Studies of Ferae-Velestinon-Rigas, 1991)
	Οne of rare books on Physics used for his enlightening against prejudices and superstitions the modern scientific knowledge of his time, which he conveyed in simple languageto be understoodby his readers; http://ebooks.edu.gr/ebooks/v/html/8547/2330/Istoria-Neoellinikis-Logotechnias_A-B-G-Gymnasiou_html-apli/index_03_02a.html
	RigasFeraios, sometimes RhegasPheraeos) or Velestinlis (1757- 1798), was a Greek writer, political thinker and revolutionary, active in the Modern Greek Enlightenment. A victim of the Balkan uprising against the Ottoman Empireand a pioneer of the Greek War of Independence,
9-MPO	Mpotsaris, 1828,MarkosMpotsaris c. 1788 –1823, was a Greekhero of the Greek War of Independence and chieftain of the Souliotes;https://www.iefimerida.gr/politismos/se-dimoprasia-epistoles-toy-mpotsari
10-KOR	Koraes (inGreek) 1816; handwritten letter of A.Koraes to K.Kokkinakis in 5 Sept. 1816; https://www.vivliothikiesiea.gr/istorika-xeirografa/
	AdamantiosKoraes or Koraïs 1748–1833 was a Greek scholar credited with laying the foundations of modern Greek literature and a major figure in the Greek Enlightenment. His activities paved the way for the Greek War of Independence
11-KOR	Koraes(in French) 1799, Letter of Koraes (signing as Coray) in French to the Italian writer Angelo Maria Bandini in Florence. In this A.Koraes informs the Italian author that he is sending him a copy of “Theophrastus” works (4th c BC, follower of Aristotle) and, among other things, asks him to announce his work in a philological magazine of his city;https://www.politischios.gr/koinonia/sto-sfyri-psifides-istorias
12-NOM	Νomarchia, 1806, earlier printed letters, cover of the Greek pamphlet ἙλληνικὴΝομαρχία (eng:HellenicNomarchy), publishedin Italy in 1806by an “anonymous Greek author”. It advocated the ideals of freedom, social justice and equality as the main principles of a well-governed society, making it the most important theoretical monument of Greek republicanism; https://en.wikipedia.org/wiki/Hellenic_Nomarchy
13-XOD	Xodilos to Xanthos,Reni, March 30, 1821; letter of AthanasiouXodilos to Emmanuel Xanthos. Published in the “EmmanouilXanthou Archive”, vol. 3, Athens, Historical and Ethnological Society of Greece, 2002; https://argolikivivliothiki.gr/2020/03/13/emmanuel-xanthos/
14-SIK	Sikelianos poet 1930, letter of A.Sikelianos to his friend F. Giophyllis from October 31, 1930; A.Sikelianoswas a famous Greek poet, one of the greatest poets of modern Greece; https://www.vivliothikiesiea.gr/istorika-xeirografa/, https://www.ekt.gr/el/copyright

All extracts from the indicated Greek magazines or journals are in Greek and are intended solely for educational and research use without any copyright restrictions but only relevant citation of the source or web site.

Moreover, it is noted that the 12-NOM is a printed text in 1806 and has its own DM that differs from others, while the two handwritten texts composed in the same year 1821 (5-KAL and 13-XOD) though slightly different may imply a similar author or a comparable handwriting style.

In such scribal investigations as described in this manuscript, all binary images have a close pixel number. The copying of images from the manuscripts should be obtained with similar, if possible, camera settings, because dilation of letters may occur that affects the fractal analysis^85^. The camera images in the investigated handwritten manuscript were taken under the same conditions. In the 13 confirmatory cases the camera setting was not controlled, and this may produce variable resolution of words which in turn may produce fuzziness at the vicinity of the text. Having this in mind we used Minkowski dimension of the letters, applying appropriate correction in cleaning (Cleaning signifies the digital process of removal some irregularities from these images because photos are captured under different conditions, paper has some oxidization and are of small size and hence exhibit some noise not belonging to the shape of the letters. By blob size threshold function in IQM automatically these artificial noises were removed.) of each letter and the words. Minkowski dimension only analyzes open-closed curves, and our examined handwriting mostly involves both open curves and closed curves.

**Figure 14:**
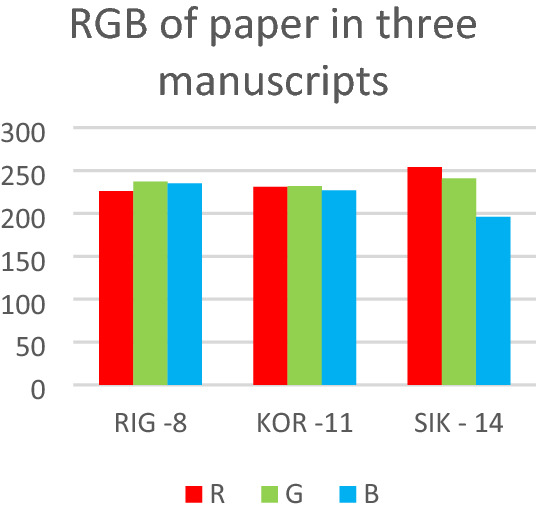
RGB of paper from three known authors handwritten manuscripts.

The study analysis of 16th and 17th century handwritten manuscripts which bear inks of various types identified as ground truth by spectroscopy was a good example to test our new RGB method for ink identification. These manuscripts were chosen only for their inks not for their handwritten analysis. The date of the 13 manuscripts were chosen on purpose around the examined manuscript age, and one of a later age, to show the differences in the D_M_ amongst different scribes and for testing the D_M_ of letters written by same authors (Koraes and Ypsilantis). Regarding the question of examining the origin of paper (production, processing, ingredients), for these manuscripts this has been tested in three cases, 8-RIG (1790), 11-KOR (1799) and 14-SIK (1930), two from the end of 18th c., and one from the beginning of 20th c. The result was encouraging in the histogram of RGB for these papers where the earlier ones are very close and quite different from the more recent one, an expected result accounting the different time of paper production techniques (Fig. 14).

## Conclusion

Novel pre-processing of images for fractal and RGB color analysis were applied to the 1821 Greek handwritten manuscript and old Slavic historical manuscripts (sixteenth -nineteenth century). The fractal Minkowski dimension used on the handwritten Greek text of initiation to the Greek *Philike Hetaereia* (Secret Society) during the Greek revolution in 1821.Analyzing five lines and full written pages identified four scribes contributing to the 20 pages of the manuscript with varying Minkowski dimensions 1.23–1.42.

RGB image analysis on the ink used and the paper for each page of the Greek manuscript also revealed **five different black iron gall inks** (four for the written text and one including first page cover design). Also, it was revealed **four scribes** from the results of the 5-lines analysis. It is of interest the striking correspondence between same pages (2, 3, 4–6, 8–18).

The novel Minkowski analysis of the full pages gave encouraging results but required each page to have been fully covered with text and the same number of lines. Four scribes are proposed for the 5-lines and at least two for all lines for the pages analysed.

The results on ink type recognition were corroborated from another Slavic manuscript whose inks were identified by Raman spectroscopy and indicating the use of various inks (black carbon, black iron gall, blue indigo, red vermillion and lead-based pigment) distinguished by RGB analysis. Last, the scribal recognition method was verified by thirteen known handwritten texts with varying Minkowski fractal dimensions from 1.20–1.52, as well as printed texts of four different languages with fractal dimensions varying between 1.30–1.38.

The qualitative and quantitative investigations of the presented methods lead to a satisfactory characterization of the different inks, and scribers, summarized by means of fingerprints. The methods take into account both the heterogeneity and the preservation status structure of historical samples. As shown in this paper, it can provide essential contributions to historical and archaeological research in a non-invasive manner, despite particle-size and minor impurity of different period inks, the ageing paper and handwriting variations.

The fundamental question of identifying scribes (i.e. names from Philike Hetaereia) for the examined manuscript is not considered, because, so far, we had no handwritten text of the fellow in the rank of “priest” Parthenopoulos or his catechist. But in the confirmatory section a similar author (Koraes and Ypsilantis) was measured by Minkowski dimension.

We are aware of the prime difficulty in handwriting identification and recognition that lies in a variety of deformation of character shapes. One can often measure differences in writing, but it is often difficult or impossible to interpret them^86^. This is subject to possible differences due various factors, such as, different writers, pen, time of day, different level of tiredness of the writer, different lighting at the time of writing, different lighting at the time of photography, different camera, and many other possibilities.

Last, but not least, the often-applied sophisticated instrumentation for the effective procedure of iron gall ink and paper characterization may be complementary non-destructive methods but require expensive laboratory infrastructure and due interpretation^71,86^.

## Material & methods

### Material

**Figure 15:**
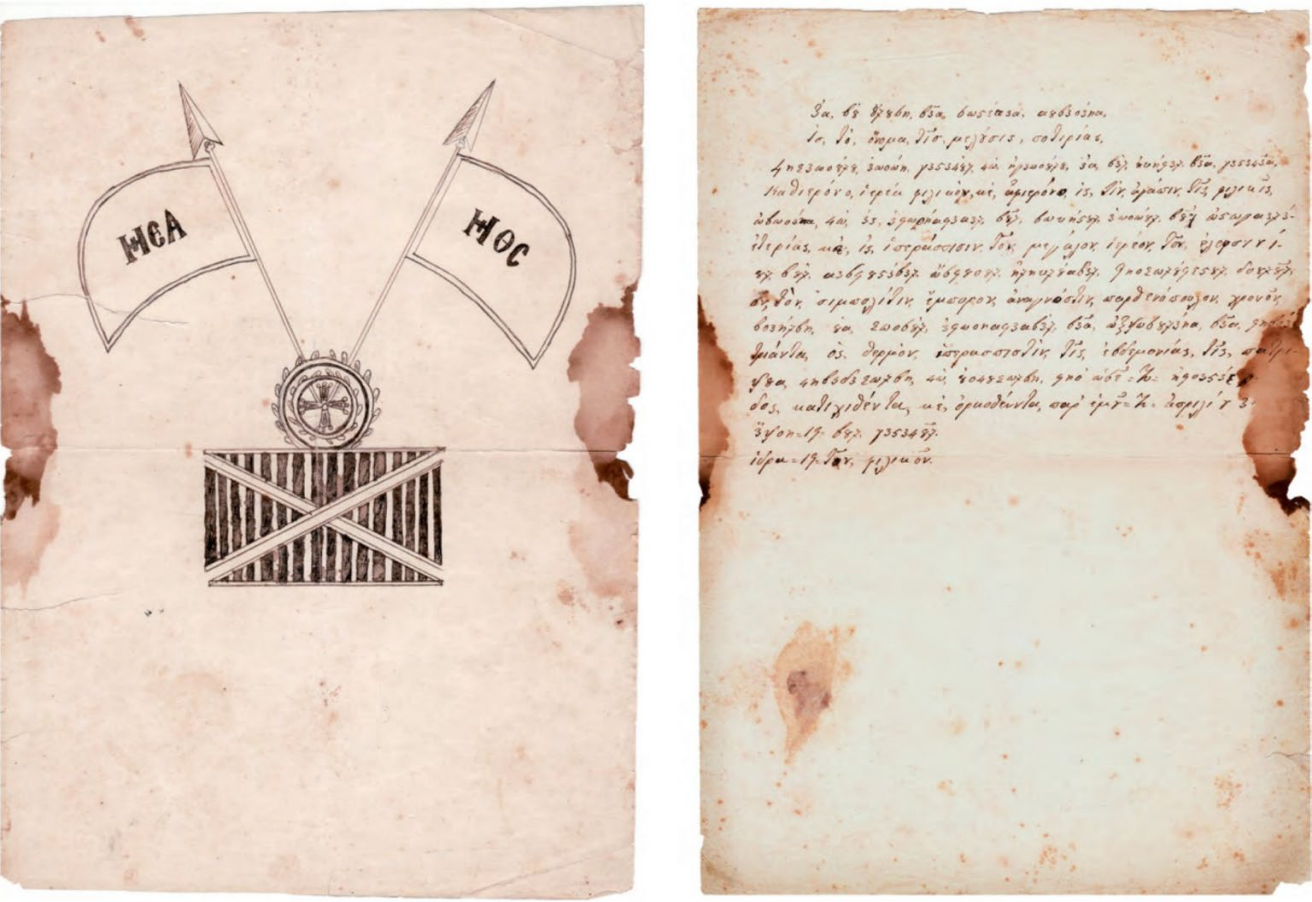
The front cover and verification letter (page 1) (Credit and permit by Grand Lodge of Greece and ^79^).

**Figure 16:**
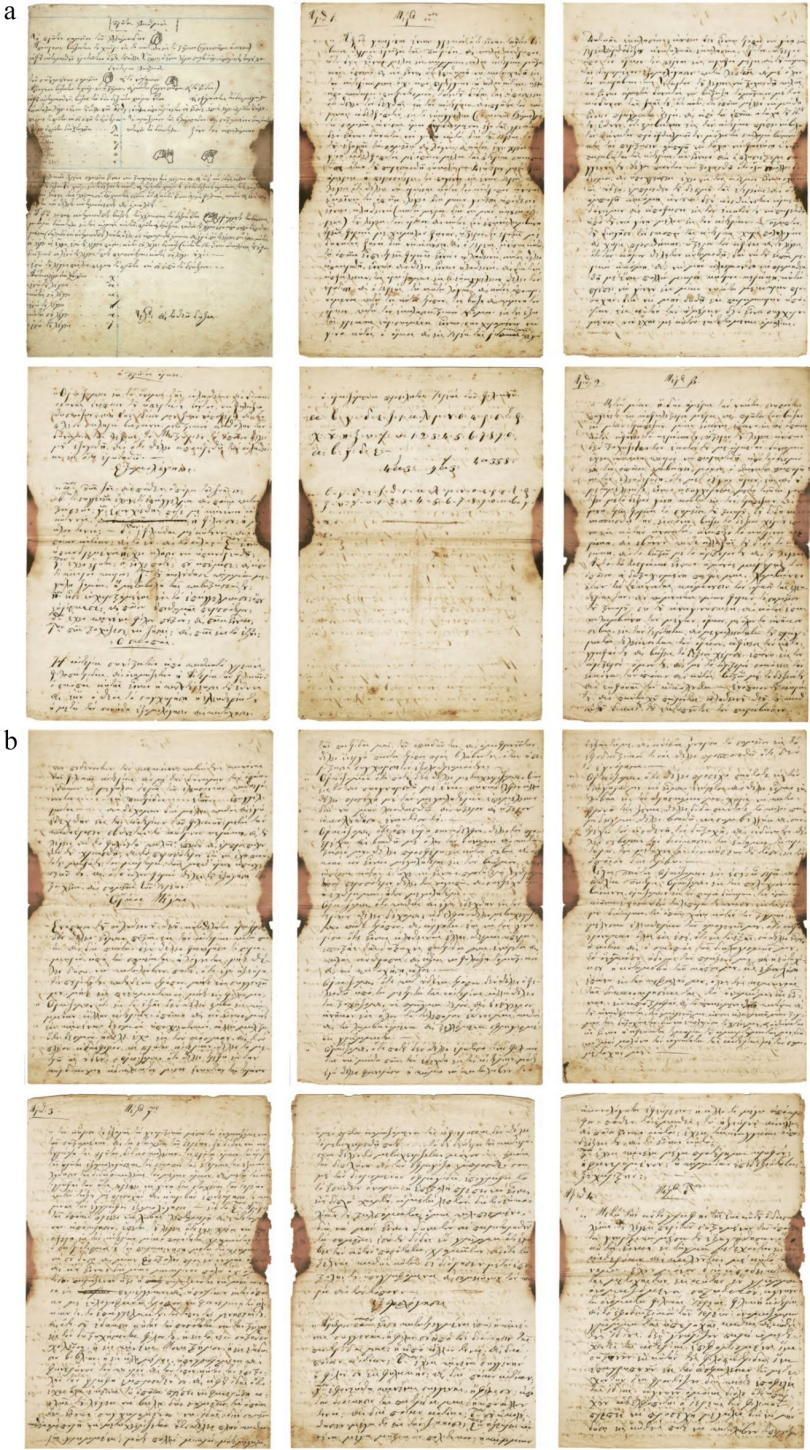

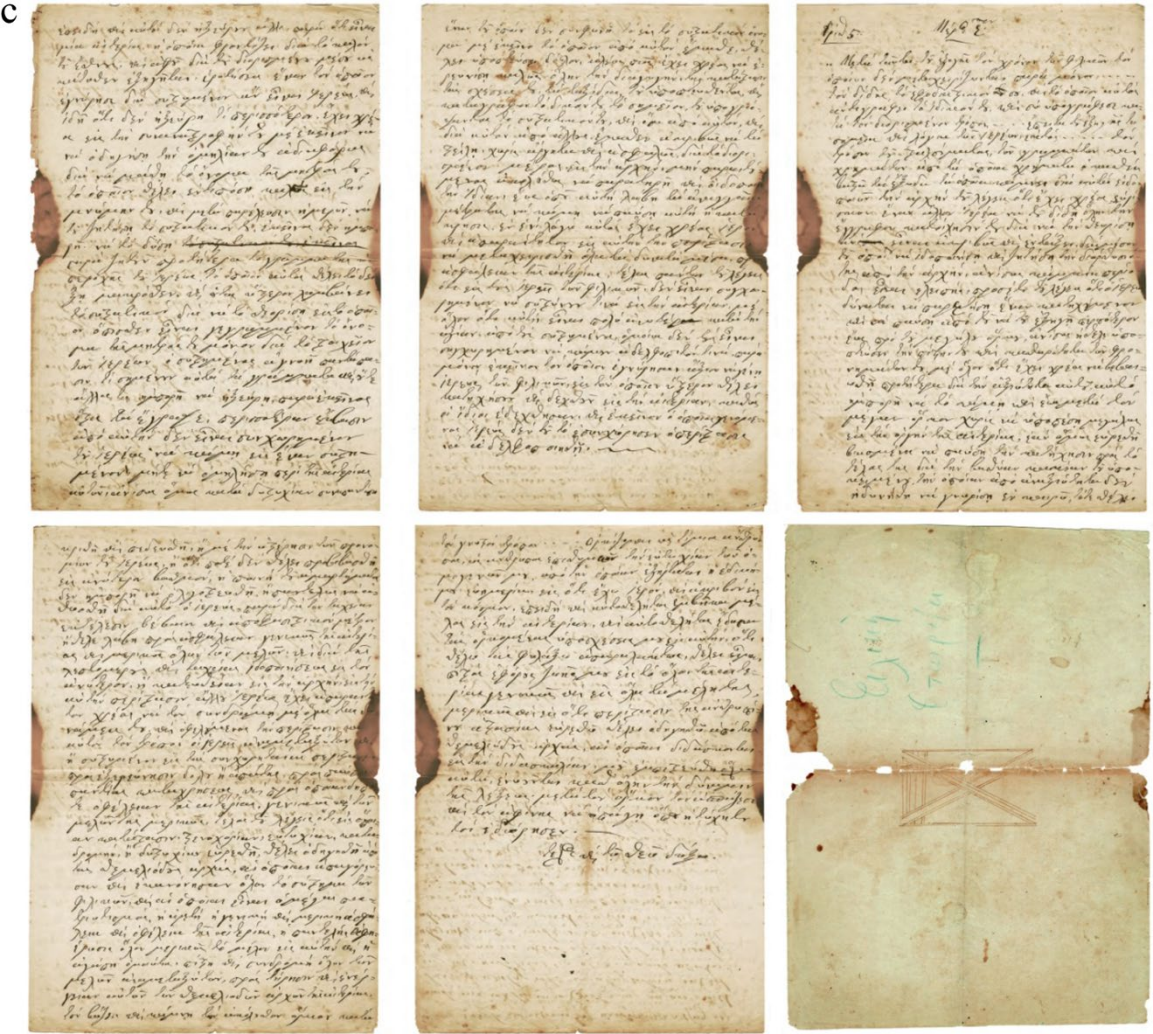
(**a**) The signs of greeting hands, the Great Oath, cryptographic alphabet and other text (pages 2–7), (**b**) more pages of text (8–13), (**c**) pages 14–18 and back cover. (Credit and permit granted by Grand Lodge of Greece and ^79^).

The 20-page A4 handwritten manuscript with front and back cover written in black ink which refers to the initiation of a new person in the “Friendly Society” was analyzed. The first page is the cover that contains wo flags and a rectangle with hatched black and white lines and the 20th page, being the back cover with a semi-finished rectangle. The second page is single folio written only in “recto” which is part of an A3 sheet, also in “recto”. The fourth to sixth pages are blank. From the seventh page onwards (in our numbering the 4th written page) the folio is written in “recto” and “verso” (first and second sides).This part of the document presents the secret initiation i.e. the written process describing how someone becomes a new member including the Society’s oath, the symbolic alphabet and a verification letter to the Fellow in the rank of “priest” of the Friendly Society, signed by the catechist, and the entire Catechism text. The uniqueness of the manuscript has been confirmed under historical investigation, and includes the name of the newcomer *Anagnostis Parthenopoulos*, which was verified in Sekeris's archive (General State Archives of Greece; http://www.gak.gr/index.php/en/)^87^ (Figs. 15, 16). The confirmation letter mentions the date of April 3, 1819 on the island of Hydra, where the initiation took place, the catechist is listed in the Sekeris archive as Nikiforos Pamboukis and the Friend who would receive it was Anagnostis Zoopoulos in Constantinople. The present initiated “Priest” is Anagnostis Parthenopoulos and the manuscript had on the cover the sacred toggle with the sixteen columns, a laurel wreath and two cross spears with hanging banners. The abbreviations “HEA” (i.e. ΗΕΛΕΥΘΕΡΙΑ/or Freedom) and “ΗΘΣ” (i.e. ΗΘΑΝΑΤΟΣ or Death) were written on the banners.

### Sampling

All analytical image processing was applied in a non-invasive manner by analyzing the text images of each page, a great advantage for analysis of precious objects.

### Methods

The use of fractal analysis and RGB image processing was applied to handwritten texts and images of letters in the text. Any scribe usually represents and reflects the writing style of the scriber. The shaping of letters and words correspond to a distinct geometrical style, reflected as a complex geometric shape of words that correspond to the fractional dimension and a pixel density distribution different to simple figures of classical, or Euclidean, geometry such as the square, circle, or sphere. Rather handwriting represents a rough or fragmented geometric shape that can be split into parts. Hence, handwriting moves beyond the four topological dimensions in traditional Euclidean geometry. The words and letters taken as iconic examples of fractals have particularities in shape along their boundaries or from the author’s style. Thus, fractal structures have either an integer dimension, or, and this is the important attribute, they possess “self-similarity/scale invariance.” Self-similar objects appear the same under magnification at all scales. They are, in some fashion, composed of smaller copies of themselves. This characteristic is often referred to as “scaling symmetry” or “scale invariance. Recently, numerical methods have been developed for estimating the dimension directly from the observed behavior of a physical system and in our present case the handwritten texts^88^. Writing is characterized by a nonlinear geometry that can be described by a non-integer dimension. Using fractal algorithms that generate visual patterns an algorithm generates a process known as recursion for the particular shapes of letters in a handwritten (or printed) text. Pre-processing of images for fractal analysis and transformation to a binary image is a pre-requisite step.

RGB analysis of images of written words with ink or the blank paper are automatically segmented, and text images are binarized and shown only with the color pixels of the ink as a histogram.

By using the Minkowski method, the relationship between the words and the area occupied by the words was analyzed. The Minkowski dimension (*D*_*M*_) is an estimation method based on the Minkowski cover, which was first examined by Bouligand (1929)^89^ using morphological dilation.In fact, the higher the value of *D*_*M*_, the closer the shape is to a perfect geometric shape. The lower the value, the more irregular the shape.*D*_*M*_ is obtained by calculating the area of the dilated object in the image^31,42^. Dilation is based on considering a circle of radius *r* that is centered at each point of the original object and all other points in the circle are connected to the object. To generate a “signature” for the shape, the area of the object is analyzed according to *r*. The algorithm used is the exact distance transformation (EDT), which represents the distance between all points in the image and the nearest point of the object. Then, the fractal dimensionis calculated by analyzing the log–log curve of the area versus *r*using the IQM 3.5 software^90^.

*D*_*M*_ is determined using the formula

$${D}_{M (r)}=2-\frac{\mathrm{log}A\left(r\right)}{\mathrm{log}r},$$

where *A(r)* is the area of influence of the object with radius *r*.

In the currentresearch, using IQM, *r*was set to 10 pixels.

### Pre-processing of images for fractal analysis

Initially images were converted from RGB to 8-bits gray-scale tiff uncompresed images. Then, the following operators were applied:

- White balance - to remove background (colour of the paper/page)
- Manual deletion of letter fragments from neighboring words
- Binarization - only words were extracted from the pages of the document
- Blob size treshold - to remove the remaining artifactsautomatically - thus full lines of the image were obtained which is suitable for fractal analysis.
-5 complete lines were extracted from full lines of the image.

All the resulted images (with 5 complete lines), were analysed with the Minkowski Dimension algorithm.

### RGB color analysis

#### Pre-processing forink color

**Figure 17:**
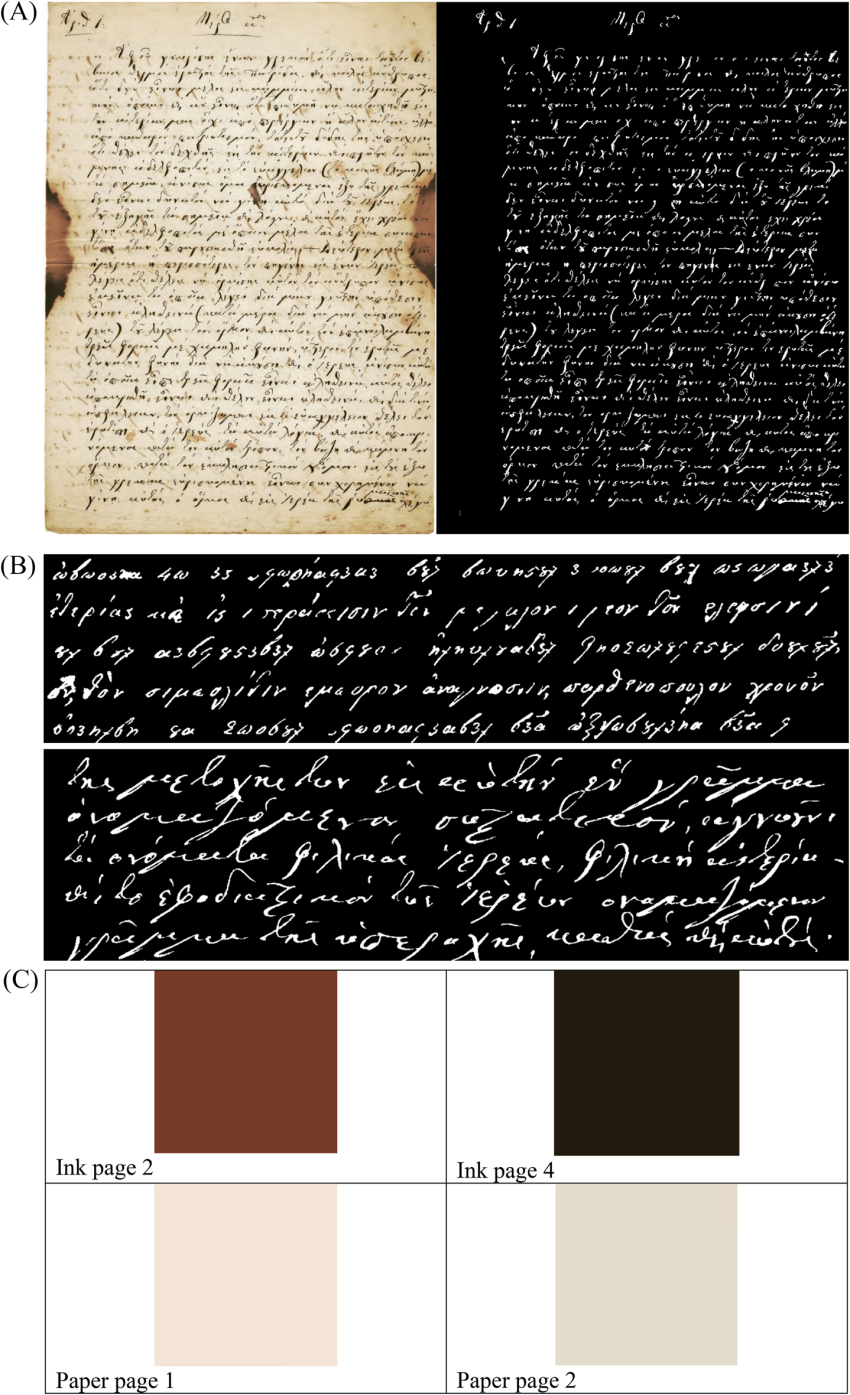

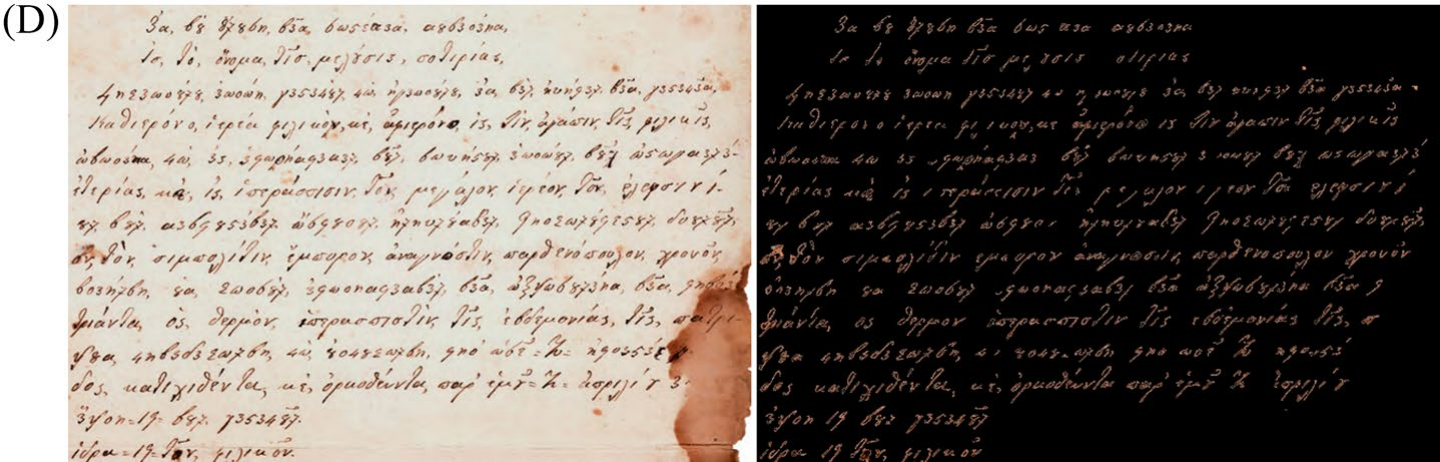
Examples of (**A**) full page 4 original (Under permission by the Grand Lodge of Greece) and binary image, (**B**) for five lines text cleaned extracts from page 2 (upper) and 14 (lower), (**C**) images of RGB value for ink of page 2 and 4 and paper of page 1 and 2, determined using IQM 3.5 and (**D**) an image with the RGB writing and black background from page 2.

Images were processed in six steps.In the first stage of the RGB images, using IQM 3.5 software the White Balance function was applied to images, with the aim of uniformizing the background, which presented some spots that would have influenced the binarization, by including them as fragments of letters. Second, the resulting images were binarized with ImageJ 1.53: white pixels representing the writing and black pixels representing the background (mask image). We used in ImageJ 1.53 the operator Make Binary default (Process - Binary - Make Binary). The threshold level was determined by analyzing the histogram of the current selection. In third step, the writing was extracted to the original RGB, and the background remained black, by applying the subtraction mathematical operator (see Fig. 17d). For this subtraction we used the Min mathematical operator, which means that pixels in the image with a value less than the specified constant are replaced by constant. The new resulting RGB image has only color pixels associated withthe ink.Fourth, RGB histogram of the image were created (only with the color pixels of the ink) using ImageJ. Next, only images where the ink chrome was dominant and darker, and occupied a central position within the letters were selected.We choose the central position of the letters, because that is where the color of the ink is preserved best. Generally, on the periphery of the letters it is possible to see more color changes either from photography / scanning (artifacts) or from the drying or ink dispersion in the paper. Finally, the pixels that met these above conditions were extracted and the RGB value determined using IQM 3.5(see below, Fig. 17c).

#### Algorithmic proccesing of words, lines, and pages

Some characteristic image examples are shown in Fig. 17, containing the 5 lines and page before and after removing noise (Fig. 17a,b).

The algorithmic processing was carried out on similar single words, on a number of lines and on the whole page of a folio. There may only be a few single words that repeat on every page, and though indicative, it is not exclusively appropriate to study the scribal. The same deficiency may be encountered in chosing one single lines per page. Therefore choosing five lines with complete text lines per page text is appropriate to identify the possible different scribes contributing to the manuscript. The whole page is also a potentially sufficient condition but not necessarily ideal if pages are not fully covered by text, and especially if the text has different spacing. In addition, any comparison the alignment and spacing at least between lines must be properly adjusted.

## Data Availability

The data used in this study is available for all researchers for non-profit purpose. All data analyzed during this study are included in google drive repository: https://drive.google.com/drive/folders/1n6bET-xk0E3l7lC6elrpUJfGHrdevQ8F?usp=sharing). Any scientific use must give credit to this article.
